# Effect of empagliflozin on left ventricular contractility and peak oxygen uptake in subjects with type 2 diabetes without heart disease: results of the EMPA-HEART trial

**DOI:** 10.1186/s12933-022-01618-1

**Published:** 2022-09-12

**Authors:** Lorenzo Nesti, Nicola Riccardo Pugliese, Paolo Sciuto, Domenico Trico, Angela Dardano, Simona Baldi, Silvia Pinnola, Iacopo Fabiani, Vitantonio Di Bello, Andrea Natali

**Affiliations:** 1 Metabolism, Nutrition, and Atherosclerosis Laboratory, Department of Clinical and Experimental Medicine, University of Pisa, Pisa, Italy; 2 Cardiopulmonary Laboratory, Department of Clinical and Experimental Medicine, University of Pisa, Pisa, Italy; 3 Department of Surgical, Medical and Molecular Pathology and Critical Care Medicine, University of Pisa, Via Savi 27, 56100 Pisa, Italy; 4 Diabetology Unit, Department of Clinical and Experimental Medicine, University of Pisa, Pisa, Italy; 5 Fondazione Toscana G. Monasterio, Pisa, Italy; 6 Pisa, Italy

**Keywords:** Empagliflozin, SGLT2, Type 2 diabetes, GLS, Speckle-tracking, Cardiovascular, Heart failure, Subclinical left ventricular dysfunction

## Abstract

**Background:**

The mechanism through which sodium-glucose cotransporter 2 inhibitors (SGLT2i) prevent the incidence of heart failure and/or affect cardiac structure and function remains unclear.

**Methods:**

The EMPA-HEART trial is aimed at verifying whether empagliflozin improves myocardial contractility (left ventricle global longitudinal strain, LV-GLS) and/or cardiopulmonary fitness (peak oxygen uptake, VO2peak) in subjects with type 2 diabetes (T2D) without heart disease. Patients with T2D, normal LV systolic function (2D-Echo EF > 50%), and no heart disease were randomized to either empagliflozin 10 mg or sitagliptin 100 mg for 6 months and underwent repeated cardiopulmonary exercise tests with echocardiography and determination of plasma biomarkers.

**Results:**

Forty-four patients completed the study, 22 per arm. Despite comparable glycaemic control, modest reductions in body weight (− 1.6; [− 2.7/− 0.5] kg, p = 0.03) and plasma uric acid (− 1.5; [− 2.3/− 0.6], p = 0.002), as well as an increase in haemoglobin (+ 0.7; [+ 0.2/+ 1.1] g/dL, p = 0.0003) were evident with empagliflozin. No difference was detectable in either LV-GLS at 1 month (empagliflozin *vs* sitagliptin: + 0.44; [− 0.10/+ 0.98]%, p = 0.11) and 6 months of therapy (+ 0.53; [− 0.56/+ 1.62]%), or in VO_*2peak*_ (+ 0.43; [− 1.4/+ 2.3] mL/min/kg, p = 0.65). With empagliflozin, the subgroup with baseline LV-GLS below the median experienced a greater increase (time*drug p < 0.05) in LV-GLS at 1 month (+ 1.22; [+ 0.31/+ 2.13]%) and 6 months (+ 2.05; [+ 1.14/+ 2.96]%), while sitagliptin induced a modest improvement in LV-GLS only at 6 months (+ 0.92; [+ 0.21/+ 0.62]%).

**Conclusions:**

Empagliflozin has neutral impact on both LV-GLS and exercise tolerance in subjects with T2D and normal left ventricular function. However, in patients with subclinical dysfunction (LV-GLS < 16.5%) it produces a rapid and sustained amelioration of LV contractility.

*Trial registration* EUDRACT Code 2016-002225-10

**Supplementary Information:**

The online version contains supplementary material available at 10.1186/s12933-022-01618-1.

## Background

In subjects with type 2 diabetes mellitus (T2D) and at high cardiovascular risk, hospitalization for heart failure (HF) were reduced by 30–40% already after 6 months of treatment with empagliflozin independently from the presence of established HF at baseline [1]. Nonetheless, the mechanisms of the cardioprotective properties that are present irrespective of the presence of T2D, the amelioration in glycaemic control, blood pressure, and body weight, remain ill-defined [2], particularly in subjects devoid of heart and kidney disease, wherein the effect on body fluid volume regulation—considered a pillar of SGLT2i mechanism of action [3]—is unlikely to play a relevant role. Alternative pharmacological actions have been suggested, namely: improved muscle oxygen/work coupling driven by a larger availability of oxygen (through increased plasma haemoglobin), the use of more efficient metabolic substrates (ketone bodies [4]), and/or a direct effect on myocardial contractility through the inhibition of the Na/H exchanger [5]. Therefore, it is possible to hypothesize that SGLT2i might exert their positive effects on primary HF prevention particularly in those with early and mild forms of left ventricular (LV) contractility dysfunction. This condition—although clinically elusive—is extremely frequent in T2D, with a prevalence ranging from 50 to 70% when more sensitive techniques such as LV global longitudinal strain (LV-GLS) by speckle-tracking echocardiography are employed [6]. Among these nominally asymptomatic subjects, a large proportion (30–45%) shows a reduced cardiopulmonary fitness [7] with complex and uncertain pathobiology that bears an increased risk of incident symptomatic HF [8].

The difficulty in accruing clinical evidence in support for these hypotheses is possibly due to the inadequacy of the experimental design and/or of the methods employed to measure cardiopulmonary function with the necessary precision. Imaging cardiopulmonary exercise test (iCPET), being a powerful multiparametric technique capable of providing simultaneous measures of metabolic, pulmonary, cardiac, muscular, and vascular variables both at rest and during graded exercise [9], qualifies as a strategic tool. CPET is particularly useful in T2D, as patients with diabetes are at increased risk of heart failure and often show exercise intolerance early in the course of the disease, before developing clinically manifest HF [8]. Similarly, signs of left ventricular systolic and diastolic dysfunction can be detected at resting conditions early in the course of the disease. However, alterations in systo-diastolic functions during exercise can be revealed even earlier in diabetic individuals who are asymptomatic at rest, with prognostic and therapeutic meaning [7, 9] as also advocated by the most recent European guidelines for the study of cardiac dysfunctions in diabetes [10].

By using iCPET, this study aimed at verifying whether the treatment with empagliflozin is associated with an improvement in cardiac systo-diastolic functions and/or in cardiopulmonary fitness in asymptomatic T2D patients without overt heart disease and normal LV ejection fraction (LVEF > 50%). To account for the potential positive effects of improved glycemic control, we used sitagliptin as an active control, an equally effective glucose-lowering agent that has been shown to be neutral on the prevention of HF-related events [11]. As pre-specified exploratory analysis, we also verified whether the effect of empagliflozin is more evident in subjects with subtle contractility impairment (reduced LV-GLS) and whether this associates with changes in plasma biomarkers of inflammation, oxidative stress, matrix remodelling, and myocyte strain and injury.

## Methods

### Rationale and study design

The EMPA-HEART trial is a phase III, open label, active-controlled, parallel groups, single center, exploratory study conducted in Pisa, Italy. This is a proof-of-concept study aiming at evaluating whether the chronic treatment with the SGLT2i empagliflozin can ameliorate myocardial and cardiopulmonary functions above and beyond its effect on glycemic control, in comparison to sitagliptin, an equally effective plasma glucose lowering agent presumably neutral on cardiac function. Outpatients with T2D of either sex, age 40–80 years, on stable metformin and/or basal insulin with suboptimal glycaemic control (HbA_1c_ 7.0–8.5%) were randomized to either Sitagliptin 100 mg or Empagliflozin 10 mg. Exclusion criteria were: (a) impaired kidney function (CK-EPI eGFR < 50 mL/min/1.76m^2^), (b) any heart disease defined as presence of clinically relevant cardiovascular symptom, cardiac or vascular disease or valvular defects, history of coronary artery disease or evidence of stress-induced ischemia, reduced (≤ 50%) 2D LV ejection fraction (LVEF), cardiac autonomic neuropathy, (c) any pulmonary, muscular, or orthopedic diseases potentially limiting exercise capacity. As pre-specified exploratory analysis, we evaluated whether the effect of the treatments on myocardial contractility differs in the subgroup of patients with more pronounced abnormalities at baseline (LV-GLS below the median) and whether there are treatment-related differences in the following plasma biomarkers: (a) inflammation: tumor necrosis factor-alpha (TNFα) and high-sensitive c-reactive protein (hsCRP); (b) oxidative stress: myeloperoxidase (MPO); (c) LV parietal stress: natriuretic peptides (BNP and NT-proBNP), pro-adrenomedullin (proADM); (d) cardiomyocyte damage: high-sensitive troponin T (hsTnT); and (e) extracellular matrix remodeling/fibrosis: procollagen (NT-PRO3). The rationale, study design, and the methods of the study have been previously described in detail [12].

### Cardiopulmonary exercise test protocol

A symptom-limited, graded, ramp exercise test was performed in the semi-supine position using a microprocessor-controlled stress cycle ergometer (Ergoline ergoselect 2000 GmbH, Germany). A 12-lead electrocardiogram and non-invasive arterial saturation and blood pressure (BP) were monitored continuously with heart rate (HR) and brachial BP measured at rest and every minute during exercise using a validated automatic device (Omron M6 Comfort, Kyoto, Japan). The expected VO_2peak_, estimated on the bases of patient age, height, weight and clinical history [13], was used to adjust the ramp increments (Watt) in order to allow all the patients to reach VO_2peak_ in 8 to 12 min. Breath-by-breath minute ventilation, carbon dioxide production (VCO_2_), and oxygen consumption (VO_2_) were measured using a dedicated cardiopulmonary test diagnostic device (Blue Cherry, Geratherm Respiratory GmbH, Germany). Patients not reaching a respiratory exchange ratio (RER) steadily > 1.0 during the exercise test were excluded from the analysis. We defined VO_2peak_ as the highest median value of the two 30-s intervals of the last minute of exercise, as previously validated [7, 14]. The peripheral extraction, that is arterio-venous oxygen difference (Δ(a-v)O_2_) was estimated indirectly with a validated method [7]. Oxygen pulse was calculated as VO_2peak_/HR and expressed both as absolute values (mL/beat per minute) and in percentage of VO_2peak_. An automatic procedure was used to detect the anaerobic threshold (AT) based on the V-slope, ventilatory equivalents and end-tidal partial pressure methods; AT was verified visually and, if necessary, recalculated [13]. The chronotropic response was estimated as the change in HR from rest to peak exercise, divided by the difference between the age-predicted maximal HR and the resting HR (i.e., HR reserve). Chronotropic incompetence was defined as the failure to achieve ≥ 80% (≥ 62% if taking β-blockers or calcium-channel blockers) of the HR reserve during exercise [15].

### Resting and exercise echocardiography

All patients underwent a comprehensive transthoracic echocardiography examination at rest (GE healthcare vivid e95, Milwaukee, WI, USA) according to the International Recommendations. As previously described [7], data collected at each stage (baseline, after 4 min, at the AT, and at peak effort) included: left ventricle (LV) and atrial (LA) volumes, stroke volume (SV), peak E-wave and A-wave velocities, tissue Doppler imaging (TDI)-derived S’ and e’ at the septal and lateral mitral annulus, tricuspid regurgitation velocity and systolic pulmonary artery pressure (sPAP), tricuspid annular plane systolic excursion (TAPSE); LV volumes and LVEF were calculated from the apical two- and four-chamber views using the modified Simpson’s rule. LV mass index (LVMi) was calculated according to current guidelines with 2D measures of LV indexed to body surface area. SV was calculated by multiplying the LV outflow tract area at rest by the LV outflow tract velocity–time integral measured by pulsed-wave Doppler during each activity level, as previously validated [7]. Cardiac output (CO) was calculated as the multiplication of SV and HR. Systemic vascular resistance (SVR) was calculated as the ratio of the peak mitral regurgitant velocity [m/s] to LV outflow tract time-velocity integral (TVI(LVOT)) [cm]. All measurements were reported as the average of three beats.

We measured global longitudinal strain (GLS) from the apical long-axis view and two- and four-chamber views, ensuring a frame rate > 50 Hz (GE healthcare EchoPAC BT 12). We reported the average values from the three apical views at rest and low-load effort, within the first 4 min of exercise, GLS was reported as the average of three beats and expressed in absolute values to improve readability. We excluded poorly tracked segments and patients were not analysed if more than one segment per view was deemed unacceptable.

### Plasma biomarkers assays

TNFα, MPO and hsCRP were measured by ELISA kits (TNF-apha Human, High sensitivity; Myeloperoxidase Human Instant and CRP Human, produced by Invitrogen by Thermo Fisher Scientific, MA, USA). hsTnT, BNP and NT-pro BNP were assayed by ECLIA methodology using commercial kits (Elecsys Troponin T hs, Elecsys BNP, Elecsys proBNP II, respectively) from Roche Diagnostics S.p.A., Milan (Italy) on the COBAS analyser e411. Mid-regional proADM and NT-PRO3 by ELISA kits (Human MR-ProADM and Human Procollagen III N-Terminal Propeptide) produced by MYBIOSOURCE, CA (USA).

### Statistical analysis

Analyses were performed using JMP Pro software version 13.2.1 (SAS Institute, Cary, NC). Values are presented as mean ± SD, or as median and interquartile range (IQR), for variables with normal and non-normal distribution, respectively. Comparisons between treatment groups were performed by the Student t-test for unpaired data for continuous variables and by the chi-square test for categorical variables. Variations from baseline to follow-up in the parameters in each of the two groups were presented as mean and [95% CI], the effect of the therapy at each follow-up assessment (1 and 6 months for LV-GLS; 6 months for the other endpoints and variables) was assessed by *t*-test on the differences from baseline and presented as mean [95% CI] and by ANOVA for repeated measure on the whole data set; considering the time*drug interaction effect. All tests were conducted at a two-sided (and when of borderline significance also one-sided) α level of 0.05.

## Results

### Baseline characteristics of the study population

According to inclusion and exclusion criteria, 106 consecutive patients were screened for the study from December 2017 to July 2020; after baseline evaluation, 37 were subsequently excluded because of definitive exclusion criteria and 13 did not participate for personal reasons. The recruitment was interrupted earlier due to lock-down imposed by COVID-19 pandemic. Fifty-six T2D subjects meeting the definitive inclusion/exclusion criteria were randomized to intervention, of which 27 were allocated to treatment with Empagliflozin 10 mg/die, and 29 to Sitagliptin 100 mg/die. During the follow-up, 3 patients abandoned the study for personal reasons and 1 patient in the Empagliflozin arm because of side effects (genital infections). At follow-up, 8 further patients were excluded because of suboptimal echocardiography images and/or incomplete or unreliable follow-up CPET data. The analysis was performed on 44 subjects, 22 in the Empagliflozin arm and 22 in the Sitagliptin arm. Patient disposition with the Consort 2010 flow diagram is shown in Additional File 1: Fig. S1.

Baseline characteristics of the study population are reported in Table 1. The population was mainly composed mainly by male adults with a relatively long duration of T2D and suboptimal glycaemic control. Lipid profile, haemoglobin, creatinine, and NT-pro-BNP showed comparable values between the groups, and no patient had peripheral artery disease (as assessed by ankle-brachial index). At baseline 2D-echoDoppler evaluation, all patients showed normal biventricular dimensions and systo-diastolic functions with no difference between the two groups (Table 1).

**Table 1:** Clinical characteristics of the study population

	All patients *(n* = *44)*	Empagliflozin *(n* = *22)*	Sitagliptin *(n* = *22)*	*p *value
**Clinical data**				
Male (n, %)	38 (86)	19 (86)	19 (86)	*ns*
Age (years)	61.7 ± 9.7	61.6 ± 9.6	61.8 ± 10.1	*ns*
Duration of diabetes (years)	9.6 ± 8.0	7.8 ± 6.9	11.1 ± 8.8	*ns*
Weight (kg)	84.6 ± 15.3	83.0 ± 13.6	83.7 ± 12.4	*ns*
BMI (kg/m^2^)	28.7 ± 5.3	27.8 ± 4.7	29.6 ± 5.7	*ns*
Mean BP (mmHg)	102.6 ± 11.5	102.9 ± 9.9	102.3 ± 13.2	*ns*
Active smokers, (n, %)	10 (23)	6 (27)	4 (18)	*ns*
Hypertension (n, %)	34 (77)	18 (81)	16 (72)	*ns*
**Baseline therapy**				
Metformin, n (%)	40 (91)	20 (91)	20 (91)	*ns*
Insulin, n (%)	11 (25)	7 (32)	4 (18)	*ns*
Statin, n (%)	32 (73)	18 (81)	14 (63)	*ns*
ACEi/ARBs, n (%)	27 (61)	16 (53)	11 (50)	*ns*
Beta-blockers, n (%)	10 (23)	5 (23)	5 (23)	*ns*
CCB, n (%)	10 (23)	6 (27)	4 (18)	*ns*
ASA, n (%)	16 (36)	4 (41)	7 (32)	*ns*
Thiazide diuretics, n (%)	5 (11)	3 (14)	2 (9)	*ns*
Furosemide, n (%)	1 (2)	0 (0)	1 (5)	*ns*
**Blood tests**				
HbA_1c_ (mmol/mol)	59.2 ± 6.4	57.8 ± 6.5	60.3 ± 6.2	*ns*
Total Cholesterol (mg/dL)	162 ± 33	159 ± 29	165 ± 38	*ns*
HDL-C (mg/dL)	48 ± 12	49 ± 13	47 ± 11	*ns*
LDL-C (mg/dL)	97 ± 26	95 ± 21	98 ± 30	*ns*
Triglycerides (mg/dL)	131 ± 57	121 ± 59	142 ± 54	*ns*
Haemoglobin (g/dL)	14.2 ± 1.3	14.1 ± 1.1	14.3 ± 1.4	*ns*
Creatinine (mg/dL)	0.89 ± 0.26	0.86 ± 0.31	0.92 ± 0.19	*ns*
eGFR (mL/min/1.73mq)	89.6 ± 17.4	91.5 ± 18.5	87.7 ± 16.5	*ns*
Uric acid (mg/dL)	5.55 ± 1.45	6.01 ± 1.60	5.10 ± 1.10	*ns*
UAlb.-UCreat.-Ratio (mg/g)	5 (0–15)	4 (0–7)	8 (4–36)	*ns*
NT-proBNP (pg/mL)	81 (27–118)	63 (28–121)	33 (16–76)	*ns*
**Vascular and pulmonary function**				
Ankle-Brachial-Index	1.16 ± 0.10	1.13 ± 1.1	1.18 ± 1.1	*ns*
VD/VT (%)	16.2 ± 4.9	16.4 ± 3.9	16.1 ± 5.2	*ns*
**2D-Echocardiography**				
EDVi (mL/m^2^)	51.5 ± 11.7	52.0 ± 12.2	51.0 ± 11.5	*ns*
LVMi (g/m^2^)	89.5 ± 17.3	89.9 ± 16.1	89.2 ± 18.9	*ns*
LAVi (mL/m^2^)	24.9 ± 7.5	24.8 ± 8.4	25.0 ± 6.8	*ns*
LVEF rest (%)	59.3 ± 4.5	60.5 ± 3.6	58.1 ± 5.1	*ns*
E/A ratio	0.90 ± 0.25	0.94 ± 0.26	0.86 ± 0.23	*ns*
E/e′ (cm/sec)	8.5 ± 2.5	8.3 ± 2.2	8.7 ± 2.7	*ns*

ACEi/ARBs, ACE inhibitors or angiotensin receptor blockers; BMI, body mass index; BP, blood pressure; CCB, calcium channel blockers; EDVi, end diastolic volume index; eGFR, estimated glomerular filtration rate; HbA1c, glycated haemoglobin; LAVi, left atrium volume index; LVEF, left ventricular ejection fraction. LVMi, left ventricular mass index; UAlb-UCreat. Ratio, spot urine albumine-to-creatinine ratio

### Changes in clinical and laboratory parameters

At 6 months follow-up, a small reduction in body weight was observed only in the empagliflozin arm, while no significant change in mean blood pressure or in resting heart rate was evident (Table 2). The two treatments produced a comparable reduction in HbA_1c_ while an increase in plasma hemoglobin and hematocrit and a reduction in plasma uric acid were observed with empagliflozin. The remaining hematologic parameters (lipids, creatinine, ACR) did not differ from baseline to follow-up in either group (Table 2).

**Table 2:** Mean changes [and 95% CI] from baseline to 6 months follow-up in clinical, biohumoral, echocardiographic, and exercise test parameters

	Empagliflozin *(n* = *22)*	Sitagliptin *(n* = *22)*	p value
**Clinical parameters**			
Weight (kg)	− 1.6 [− 2.7/− 0.5]*	0.1 [− 1.1/1.2]	*0.0315*
HR at rest (beat/min)	0.6 [− 1.6/2.8]	− 0.4 [− 4.5/3.7]	*ns*
MAP rest (mmHg)	− 5.4 [− 10.7/0.0]	− 0.22 [− 7.6/7.2]	*ns*
**Biohumoral parameters**			
HbA_1c_ (mmol/mol)	− 4.6 [− 7.4/− 1.8]*	− 4.9 [− 8.8/− 0.9]*	*ns*
Total Cholesterol (mg/dL)	− 8 [− 21/5]	− 15 [− 30/0]	*ns*
HDL-Cholesterol (mg/dL)	1.3 [− 1.4/4.0]	− 1.7 [− 4.2/0.9]	*ns*
LDL-Cholesterol (mg/dL)	− 7 [− 19/6]	− 7 [− 18/3]	*ns*
Triglycerides (mg/dL)	− 2 [− 28/24]	− 14 [− 33/6]	*ns*
Haemoglobin (g/dL)	0.7 [0.2/1.1]*	− 0.5 [− 1/− 0.1]	*0.0003*
Haematocrit (%)	2.0 [0.7/3.2]*	− 1.3 [− 2.6/0.0]	*0.0006*
Creatinine (mg/dL)	− 0.1 [− 0.2/0.1]	− 0.0 [− 0.1/0.0]	*ns*
eGFR (mL/min/1.73mq)	2.5 [− 3.7/8.7]	1.4 [− 1.8/4.6]	*ns*
Uric acid (mg/dL)	− 1.5 [− 2.3/− 0.6]*	0.2 [− 0.3/0.6]	*0.0023*
UAlb-UCreat-Ratio (mg/g)	6.1 [− 1.9/14.2]	5.0 [− 20.6/30.5]	*ns*
**Echocardiography**			
EDVi rest (mL/m^2^)	2.2 [− 0.9/5.2]	3.6 [− 1.0/6.3]	*ns*
LVMi rest (g/m^2^)	4.5 [− 1.1/10.2]	1.1 [− 2.7/5.0]	*ns*
LAVi rest (mL/m^2^)	0.5 [− 1.3/2.2]	2.0 [− 0.4/4.3]	*ns*
CO rest, L/min	0.0 [− 0.6/0.6]	0.8 [− 0.3/1.4]	*ns*
CO peak, L/min	0.7 [− 0.6/1.9]	0.9 [− 0.3/2.1]	*ns*
LVEF rest (%)	0.1 [− 1.3/1.6]	2.1 [− 0.4/3.7]	*ns*
LVEF peak (%)	− 0.7 [− 2.8/1.5]	2.0 [− 0.1/3.9]	*ns*
S’ mean rest (cm/sec)	0.0 [− 0.8/0.9]	− 0.1 [− 1.0/0.8]	*ns*
S’ mean peak (cm/sec)	0.4 [− 0.9/1.7]	− 0.2 [− 1.0/0.6]	*ns*
ΔS’ mean	0.4 [− 0.8/1.5]	− 0.1 [− 1.0/0.8]	*ns*
E/e’ rest (cm/sec)	− 0.5 [− 1.3/0.4]	− 1.0 [− 2.2/0.2]	*ns*
E/e’ peak (cm/sec)	− 0.3 [− 1.5/0.9]	− 0.6 [− 1.5/0.5]	*ns*
**Cardiopulmonary exercise test**			
Workload (W)	5 [− 1/11]	2 [− 5/9]	*ns*
HR at peak (beat/min)	3.0 [− 2.1/8.0]	1.3 [− 4.4/7.0]	*ns*
HR at peak (%max)	1.9 [− 1.3/5.1]	0.8 [− 2.8/4.5]	*ns*
RER peak	0.00 [− 0.03/0.04]	0.01 [− 0.02/0.03]	*ns*
VO_2_/work slope	0.3 [− 0.5/1.1]	0.6 [− 0.2/1.4]	*ns*
VO_2_ rest (mL/min/kg)	0.5 [− 0.1/1.2]	0.6 [− 0.1/1.4]	*ns*
VE/VCO2 slope	0.3 [− 1.2/1.8]	1.3 [− 0.1/2.6]	*ns*
O_2_ pulse peak (mL/bpm)	0.1 [− 0.7/1.0]	0.5 [− 0.2/1.2]	*ns*
O_2_ pulse peak (%VO_2_peak)	2.8 [− 3.0/8.5]	3.0 [− 1.1/7.1]	*ns*
AV O_2_ diff rest (mL/dL)	0.6 [− 0.7/1.8]	0.2 [− 1.0/1.4]	*ns*
AV O_2_ diff peak (mL/dL)	− 0.1 [− 0.9/0.7]	− 0.2 [− 1.3/1.0]	*ns*

*Indicates a statistically significant difference within groups, p value indicates the level of statistical significance of the interaction term time*treatment at MANOVA

### Resting and exercise echocardiography

**Fig. 1:**
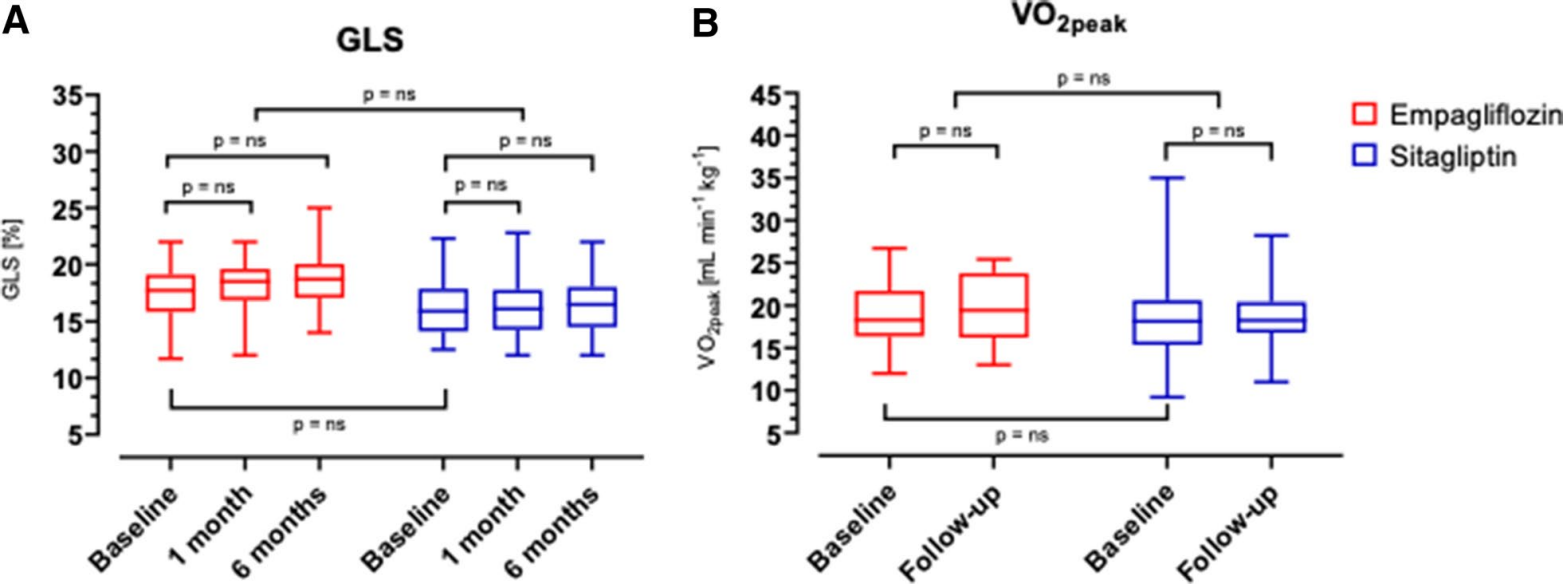
Box-and-whiskers plots of **a** left ventricle global longitudinal strain (GLS) and **b** oxygen uptake at peak exercise (VO_2peak_) at baseline evaluation and at follow-up visits, expressed in absolute values (% and mL/min/kg, respectively)

At baseline, resting and effort indices of heart function were similar in the two study groups (Additional file 2: Table S1); baseline resting LV-GLS was numerically higher in the empagliflozin group (17.3 ± 2.7 *vs* 15.8 ± 2.2%, p = 0.06). From baseline to 1- and 6-months follow-up, no change in resting LV-GLS was seen in any of the treatment groups (Fig. 1A); the difference between the treatments was slightly in favour of empagliflozin both at 1 month (+ 0.44 [− 0.10/+ 0.98]%) and at 6 months (+ 0.53 [− 0.56/+ 1.62]%); however, the time*drug effect at ANOVA for repeated measures was not statistically significant. The exercise-induced acute increase (from rest to 4 min of exercise) in LV-GLS was comparable in the two treatment arms both at baseline (+ 1.9 [+ 1.1/+ 2.6] *vs* + 1.9 [+ 1.2/+ 2.5]% for empagliflozin and sitagliptin, respectively) and at 6 months follow-up (+ 1.4 [+ 0.6/+ 2.1] *vs* + 2.0 [+ 1.2/+ 2.7]%). Likewise, cardiac chamber dimensions and/or geometry were not affected by either treatment, as well as Doppler and tissue-Doppler derived systo-diastolic indices (LA volume index, LVEF, LV mass index, E/A ratio, mitral anulus S’, e’, E/e’, TAPSE, sPAP) and SVR (Table 2).

### Cardiopulmonary exercise test

At baseline, indices cardiopulmonary function were similar in the two study groups (Additional file 2: Table S1). All patients reached a maximal exercise as required by inclusion criteria, achieving a respiratory exchange ratio (RER) steadily above 1.0 (median: 1.07, IQR: [1.03–1.10]), and the duration of exercise was between 10 and 12 min as per protocol. The exercise was well tolerated without discomfort, hypertensive response, or any significant alteration in vital parameters or ECG trace. The achieved VO_2peak_ at baseline in the whole population was 18.9 [15.8–21.3] mL/kg/min, which corresponded to 76 ± 15% of predicted maximal theoretical VO_2_ and was similar in the two groups (empagliflozin 18.9 ± 3.8 *vs* sitagliptin 18.8 ± 5.6 mL/min/kg), as was comparable the achieved peak workload (118 ± 25 *vs* 119 ± 22 W). From baseline to 6 months follow-up, no change in VO_2peak_ was seen in any of the treatment groups (Fig. 1B). Also, we could not demonstrate any variation from baseline in each treatment arm or between the arms in the other main parameters derived from iCPET, namely: cardiac (cardiac output, chronotropic response, oxygen pulse), pulmonary (ventilatory efficiency, oxygen saturation, end-tidal carbon dioxide), skeletal muscle (peripheral oxygen extraction), and metabolic (RER, anaerobic threshold). The results are reported in Table 2.

### Plasma biomarkers

The baseline plasma levels of the biomarkers (median [IQR]) were within the normal range and no change was observed at 6 months follow-up in either treatment arm or between the treatments. Specifically: hsCRP (mg/dL) from 0.114 [0.026–0.216] to 0.095 [0.048–0.153] with empagliflozin, from 0.177 [0.090–0.762] to 0.156 [0.081–0.405] with sitagliptin. BNP (pg/mL) from 25 [10–47] to 16 [10–46] with empagliflozin, from 12 [10–25] to 11 [10–20] with sitagliptin. TnHS (ng/mL) from 9.9 [6.7–16.4] to 10.0 [7.6–13.9] with empagliflozin, from 7.8 [5.8–27.0] to 8.2 [6.6–15.2] with sitaglitpin. ProADM (nmol/L) from 0.080 ± 0.065 to 0.150 ± 0.120 with empagliflozin, from 0.154 ± 0.198 to 0.119 ± 0.130 with sitagliptin. NT-PRO3 (ng/mL) from 5.6 [4.4–6.7] to 5.6 [4.0–7.7] with empagliflozin, from 6.7 [5.1–8.9] to 6.2 [4.7–7.6] with sitagliptin. TNFα (pg/mL) from 0.74 [0.46–0.96] to 0.79 [0.69–0.96] with empagliflozin, from 0.67 [0.59–0.88] to 0.80 [0.66–0.93] with sitagliptin. All p values > 0.05.

### Subgroup analysis

**Fig. 2:**
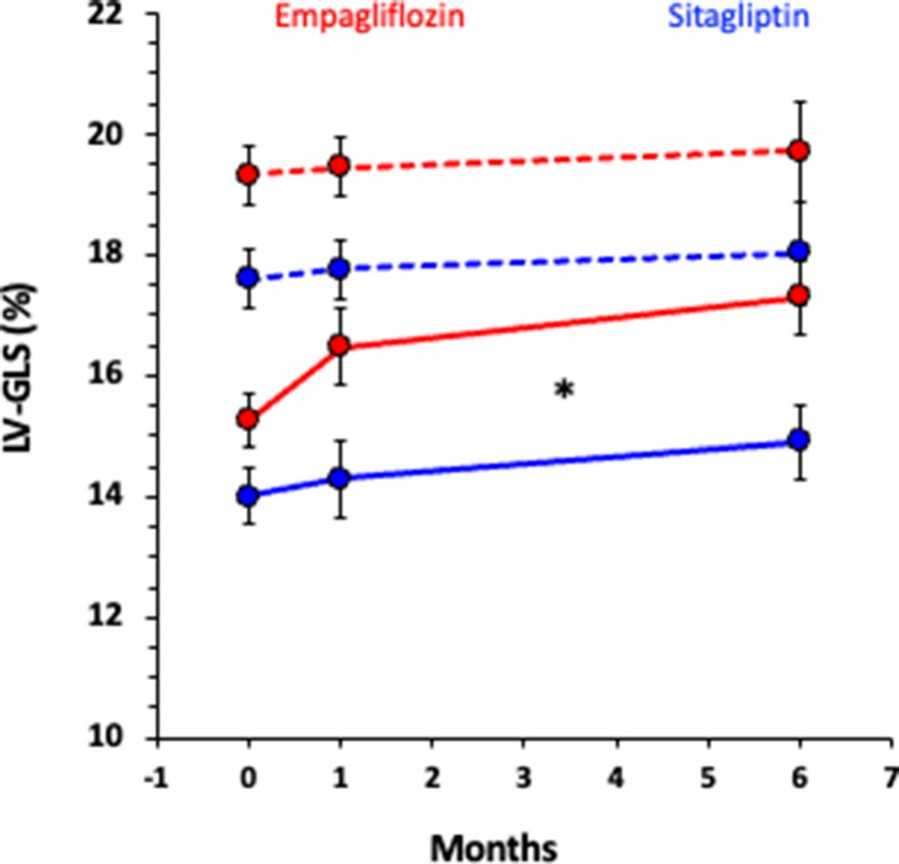
Values of left ventricle global longitudinal strain (GLS) at baseline (0), 1 month and 6 months follow-up visits during empagliflozin (red) or sitagliptin (blue) treatment. The population was divided in two subgroups depending on baseline GLS values above (continuous line) or below (dotted lines) median (median for empagliflozin group: GLS 16.4%; median for sitagliptin group: GLS 16.0%). The star indicates a statistically significant time*treatment effect at *ANOVA for repeated measures*

As prespecified hypothesis-driven analysis, we divided each arm in two subgroups of 11 subjects according to the ranking of baseline resting LV-GLS values (median GLS empagliflozin 16.5%, median GLS sitagliptin 16.0%). The subgroups with higher LV-GLS showed no change during the study neither on empagliflozin nor sitagliptin. On the contrary, the subjects with lower baseline LV-GLS experienced an improvement in LV contractility absolute values already at 1 month after therapy with empagliflozin (+ 1.22 [+ 0.31/+ 2.13]%) followed by a further improvement at 6 months (+ 2.05 [+ 1.14/+ 2.96]%). The subjects with lower LV-GLS on sitagliptin showed no change at 1 month (+ 0.30 [− 0.13/+ 0.73]%) and a mild improvement at 6 months (+ 0.92 [+ 0.21/+ 0.62]%) (Fig. 2). The estimated differences between the changes induced by the 2 treatments by paired *t*-test were + 0.92 [− 0.04/+ 1.89]% (p = 0.05 for 2-side and p = 0.03 for one-side superiority of empagliflozin) at 1 month and was maintained at 6 months (+ 1.08 [+ 0.01/+ 2.17]%, p = 0.05 for 2-side and p = 0.03 for one-side superiority of empagliflozin). The ANOVA for repeated measures detected a significant effect for the interaction term time*drug (p = 0.04) as well as for the *drug* (p = 0.02) and for *time* (p < 0.0001) alone.

## Discussion

The EMPA-HEART is a randomized trial aimed at evaluating whether the treatment with empagliflozin is associated with an amelioration of LV contractility and/or cardiopulmonary function independently of its effects on glycaemic control in T2D subjects without clinical or echocardiographic evidence of cardiac disease. In line with previous observations [16, 17], the treatment with empagliflozin was associated with a modest reduction in body weight and serum uric acid, as well as to an increase in hemoglobin and hematocrit.

No significant change in structural parameters were appreciated at resting 2D echocardiography; similarly, despite a trend towards improved values, diastolic function was also unchanged in the two intervention groups, both at rest and during exercise. SGLT2i have been inconsistently associated with an amelioration of LV structural and functional parameters in T2D subjects without overt HF and/or structural heart disease[2]. Sitagliptin has been shown to improve E/e′ by 20% in a population similar to ours, but only after 24 months [18], and empagliflozin has been reported to ameliorate diastolic function in subject with HFrEF and moderate to severe diastolic dysfunction [19]. Correspondingly, the modest reduction in LVMi reported by one uncontrolled trial [20] was not evident in our study.

With regard to systolic parameters, the crude indices provided by resting and exercise 2D LVEF did not change significantly at follow-up neither in the whole population nor in any of the treatment arms, confirming the unimportant effect of SGLT2i on this parameter in subjects without heart disease [2]. This is corroborated by the unchanged tissue Doppler S’ and speckle-tracking LV-GLS values, more sensible and less load-dependent systolic parameters than 2D LVEF (Fig. 1 and Table 2). Nevertheless, when considering subgroup analysis, while no change was observed in those with higher LV-GLS values, patients with subclinical LV contractile dysfunction (LV-GLS < 16.5%) on empagliflozin showed a significant increase in LV-GLS at follow-ups, that was evident already at 1 month and further improved at 6 months. On the contrary, in the sitagliptin arm the increase in contractility in the subgroup with lower baseline LV-GLS (< 16.0%) was evident only at 6 months and was approximately 50% smaller (Fig. 2). The similarity between the change in GLS from 1 to 6 months in both treatment groups suggests that glycaemic control per se might have had a favourable effect on myocardial contractility, as it has been recently suggested [21]. Our results imply that empagliflozin can improve LV contractility beyond its glycaemic effects in those with subclinical myocardial dysfunction. A recent publication with cardiac magnetic resonance supports this interpretation [22]. The cut-off value that we identified for a benefit during SGLT2i therapy is in accordance with a recent definition of normal LV-GLS in adults as > 18%, borderline values 16–18% and abnormal as < 16% [23].

It is known from the literature that SGLT2i are associated with a relatively heterogeneous amelioration of LV-GLS (from 2 to 11% over baseline values) despite no increase in 2D-LVEF in subjects with T2D and HF with a gradient that is proportional to the degree of baseline dysfunction [24, 25]. A 12-months long, randomized, open label clinical trial reported no effect on LV-GLS after treatment with SGLT2i (LV-GLS 17 ± 4 *vs* 17 ± 4%) in 40 subject with T2D, normal LVEF, and no clinical diagnosis of HF [26]; unfortunately, subgroup analysis according to baseline GLS was not performed in that study. Our results in a similar population extend the concept that empagliflozin ameliorates systolic function in T2D in proportion to baseline values [2] to include also those with early and mild subclinical contractility impairment in the absence of overt cardiac disease. Our data also indirectly confirm the high prevalence of subclinical contractility dysfunction reported in the asymptomatic T2D population (approx. 50%) [6]. Considering the prognostic value of LV-GLS [27], our finding might represent a solid rationale for verifying through a randomized double blind clinical trial whether the early use of empagliflozin can prevent or delay incident HF in this specific subgroup of patients, currently not specifically included in guidelines on the use of SGLT2i in HF prevention in T2D.

Since in T2D the condition of reduced VO_2peak_ is associated with adverse cardiovascular outcomes [28], one may postulate that an increased cardiopulmonary function might be observed with SGLT2i therapy. In previous pilot studies lacking randomization and active control, VO_2peak_ was increased by 24% after 6 months of therapy with empagliflozin *vs* “usual therapy” in T2D patients with established cardiovascular disease or at high risk [29], and by 10% in HFrEF patients with [30] and without T2D [31] after 1 month of therapy. Conversely, more rigorous studies in T2D and HFrEF failed to substantiate any improvement after SGLT2i either alone [32] or versus an active control [33]. In our study, cardiopulmonary fitness and all the major parameters influencing VO_2peak_—i.e., cardiac output, peripheral extraction, ventilation—were unaffected by either treatment, further sustaining the observations of a neutral effect of either drug on cardiopulmonary capacity in this population. The amelioration of glycaemic control is known to improve VO_*2peak*_ in T2D with established cardiac disease [34, 35] and can justify the positive results of the non-controlled, non-randomized trials that were not confirmed when active controls were used, as it is in the present study. Interestingly, the subjects with HFrEF and concomitant therapy with loop diuretics showed a greater improvement in cardiorespiratory fitness when receiving empagliflozin [32] and this implies a synergism between the two diuretics in volume regulation as elegantly shown by Griffin et al. [36]. No patient was assuming loop diuretics in our population, and this could partly justify the negative results on VO_2peak_, which on the other hand confirms that volume regulation is unlikely to be the mechanism through which SGLT2i are effective in primary prevention (i.e., in patients with no congestion). We have recently shown that both effort tolerance (VO_2*peak*_) and peripheral oxygen extraction are correlated with LV contractility indices (S′ and GLS) in subjects with uncomplicated T2D [7] suggesting the presence of a subclinical myopathy involving both the heart and the skeletal muscle. Accordingly, in the present study, VO_*2peak*_ values showed a trend to be lower in those with LV-GLS below the median (17.5 ± 1.0 *vs* 19.9 ± 1.0 mL/min/kg, p = 0.12). Nonetheless, our result of unchanged peak workload and peripheral oxygen extraction confirms the lack of clinically relevant effects of SGLT2i on skeletal muscle oxygen/work coupling in T2D subjects.

No significant change in natriuretic peptides was evident from our data, which were in the normal reference values at baseline. This confirms that volume regulation is not relevant in this study population, aligning with the available literature that failed at demonstrating a consistent reduction in natriuretic peptides with SGLT2i, with a trend towards a greater efficacy in patients with HFrEF [24] and higher baseline values of natriuretic peptides [2]. Differently from pre-clinical evidence of anti-inflammatory [37] and anti-fibrotic properties of SGLT2i [38], in this study the markers of myocardial injury, oxidative stress, matrix remodelling, and inflammation were unchanged at follow-up. Still, the neutral effect on biomarkers of matrix remodelling agrees with a recent study with cardiac magnetic resonance imaging detecting no change in myocardial fibrosis after empagliflozin therapy in T2D subjects with diabetic cardiomyopathy [39].

## Conclusions

In T2D subjects free from heart disease empagliflozin has a neutral impact on aerobic fitness and LV systo-diastolic functions, both at rest and during exercise. Nevertheless, it can exert an early and sustained amelioration of myocardial contractility in those with subclinical dysfunction as defined by a mildly reduced resting LV-GLS (< 16.5%) despite normal LVEF. These data support the hypothesis that SGLT2i can directly affect myocardial contractility in selected patients with subclinical LV systolic dysfunction, possibly justifying their benefit in HF primary prevention.

## Limitations

The recruitment was interrupted early due to the lock-down imposed by the COVID-19 pandemics, therefore the power of our study is lower than planned; therefore, we might have missed absolute changes in LV-GLS below 2.5% or 1.7%, which were considered relevant from a clinical and pathophysiologic point of view, respectively [12]. The data, however, are clear in showing no change in LV-GLS in each group despite a clinically relevant change (+ 2.05 [+ 1.14/+ 2.96]%) in the subjects with low baseline LV-GLS treated with empagliflozin for 6 months. The reduced sample size also forced us to restrict the secondary endpoints to only one (VO_2peak_) and the pre-defined exploratory analysis only to subgroup analysis according to baseline LV-GLS and to mechanism-oriented biomarkers. The *a posteriori* power calculation on LV-GLS measured on 44 (22 per arm) subjects, with paired comparison within each group, showed statistical power between 99 and 84% for differences ranging from 2.5 to 1.7% and the smallest difference in LV-GLS—keeping power at 95%—is 2.0%. Therefore, we acknowledge that changes smaller than 2% might be missed because of the reduced sample size. This value still is close to 1.5%, our definition of minimal clinically meaningful change. Although LV-GLS is considered an accurate method to evaluate LV contractility, there is evidence that it might be affected by the technology used, age, sex, BMI, and to some extent also by LV loading conditions [40]. In our study all these variables remained stable; therefore, while the absolute values might be difficult to interpret, the changes within subjects are robust. This was an open study, but the cardiologist performing the measurements of primary and secondary outcomes was blind to the treatment allocation.

## Supplementary Information

**Additional file 1: Figure S1.** EMPA-HEART trial.

**Additional file 2: Table S1.** Cardiopulmonary exercise test, 2D Echocardiograpy, Doppler, Tissue Doppler, and Speckle tracking parameters. P-value is the result of a two-point ANOVA for repeated measures.

## Data Availability

The study was carried out in accordance with the most recent international GCP guidelines (CPMP/ICH/135/1995), EU Directive and guidance and the local legislation on the conduct of clinical trials. The database has been locked after the planned statistical analysis was performed. Any further modification of recorded data will be documented in a database log form. All the collected data will be stored for a maximum period of 15 years after the end of the study and then destroyed. Only the investigators of the study will have access to all the data.

## Abbreviations

AT: Anaerobic threshold
BMI: Body mass index
BNP: Brain-derived natriuretic peptide
BP: Blood pressure
CO: Cardiac output
eGFR: Estimated glomerular filtration rate
HbA_1c_: Glycated hemoglobin
HF: Heart failure
HFpEF: Heart failure with preserved left ventricular ejection fraction
HFrEF: Heart failure with reduced left ventricular ejection fraction
HR: Heart rate
hsCRP: High-sensitive C-reactive protein
hsTnT: High-sensitivity troponin T
iCPET: Imaging cardiopulmonary exercise test
LA: Left atrium
LV: Left ventricle
LV-GLS: Left ventricle global longitudinal strain
LVEF: Left ventricular ejection fraction
LVMi: Left ventricular mass index
MAP: Mean arterial pressure
MPO: Myeloperoxidase;
NT-PRO3: N-terminal procollagen 3
NT-proBNP: N-terminal-proBNP
proADM: Pro-adrenomedullin
RER: Respiratory exchange ratio
SGLT2i: Sodium-glucose co transporter 2 inhibitors;
sPAP: Systolic pulmonary artery pressure
SV: Stroke volume
SVR: Systemic vascular resistance
T2D: Type 2 Diabetes;
TAPSE: Tricuspid anulus plane systolic excursion
TNF-α: Tumor necrosis factor-alpha;
VCO_2_: Carbon dioxide production
VD/VT: Dead volume/tidal volume radio
VE: Minute ventilation
VE/VCO_2_: Ventilatory efficiency
VO_2_: Oxygen uptake
VO_2peak_: Oxygen uptake at peak exercise
Δ(a-v)O_2_: Artero-venous oxygen difference (peripheral extraction)
