# Supplementary figures and images for: Effect of empagliflozin on left ventricular contractility and peak oxygen uptake in subjects with type 2 diabetes without heart disease: results of the EMPA-HEART trial
Source: Cardiovasc Diabetol. 2022 Sep 12;21:181. doi: 10.1186/s12933-022-01618-1 (PMC9467417; doi:10.1186/s12933-022-01618-1)

# EMPA-HEART trial

## CONSORT 2010 Flow Diagram

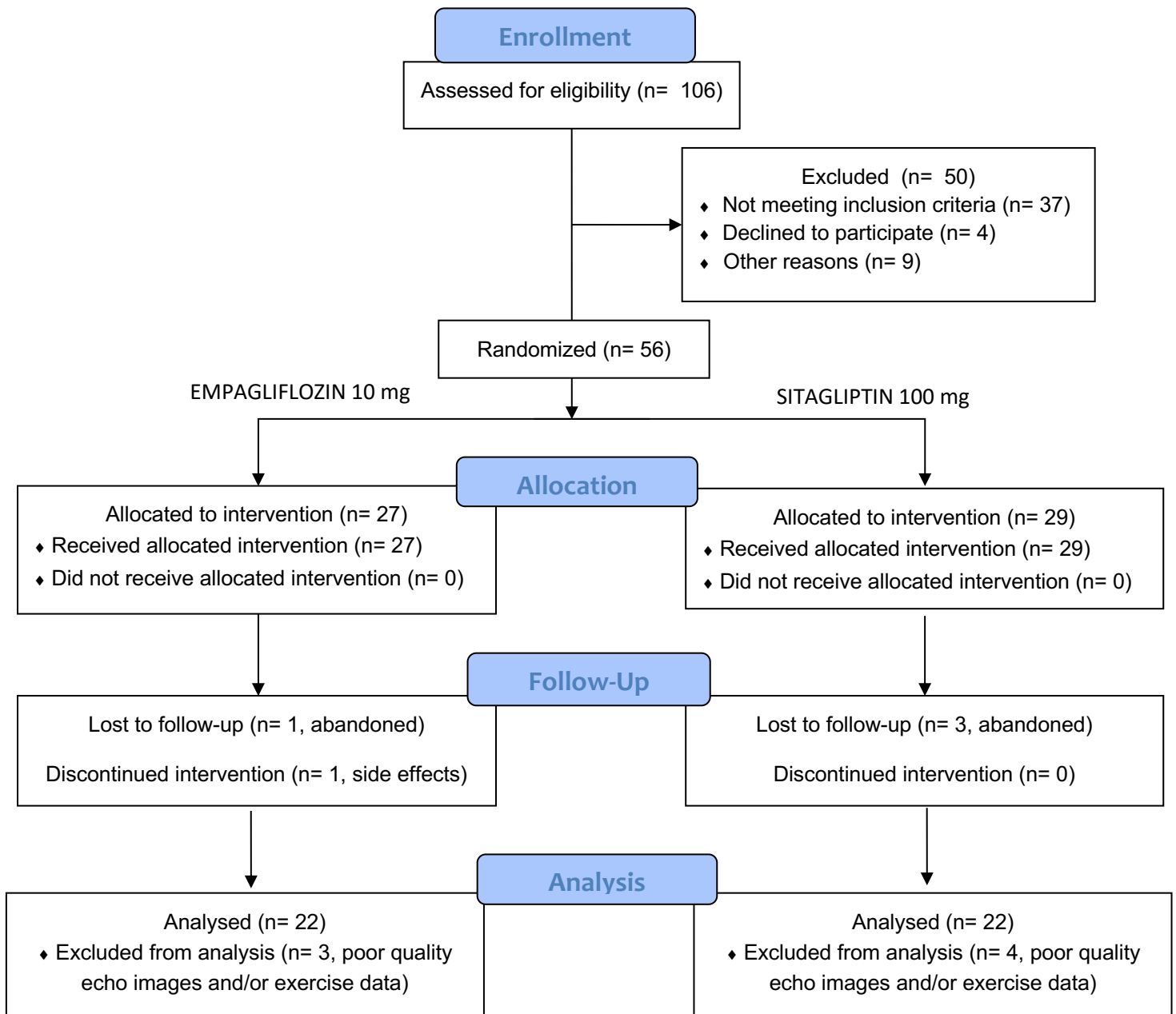

Supplement: Supplementary file 1 — Additional file 1: Figure S1. EMPA-HEART trial. [file 12933_2022_1618_MOESM1_ESM.pdf]
