# Supplementary material for: Effect of empagliflozin on left ventricular contractility and peak oxygen uptake in subjects with type 2 diabetes without heart disease: results of the EMPA-HEART trial
Source: Cardiovasc Diabetol. 2022 Sep 12;21:181. doi: 10.1186/s12933-022-01618-1 (PMC9467417; doi:10.1186/s12933-022-01618-1)
Supplement: Supplementary file 2 — Additional file 2: Table S1. Cardiopulmonary exercise test, 2D Echocardiograpy, Doppler, Tissue Doppler, and Speckle tracking parameters. P-value is the result of a two-point ANOVA for repeated measures. [file 12933_2022_1618_MOESM2_ESM.docx]

|  | Empagliflozin  *(n = 22)* | | Sitagliptin  *(n = 22)* | |  |
| --- | --- | --- | --- | --- | --- |
|  | **Baseline** | **Follow-up** | **Baseline** | **Follow-up** | **p value** |
| Cardiopulmonary exercise test | | | | | |
| *Workload (W)* | 118 ± 26 | 123 ± 31 | 119 ± 32 | 121 ± 29 | ns |
| *Time of effort (min)* | 11.3 ± 2.2 | 11.4 ± 1.7 | 11.0 ± 2.3 | 11.0 ± 1.7 | ns |
| *HR at rest (beat/min)* | 76.5 ± 11.6 | 77.1 ± 11.4 | 80.2 ± 13.4 | 79.8 ± 10.8 | ns |
| *HR at peak (beat/min)* | 129.1 ± 15.6 | 132.1 ± 14.3 | 133.6 ± 19.6 | 134.9 ± 18.8 | ns |
| *HR at peak (%max)* | 83.2 ± 9.4 | 85.1 ± 9.1 | 86.2 ± 11.9 | 87.0 ± 11.4 | ns |
| *Chronotropic incomp (n, %)* | 16 (57) | 15 (54) | 12 (43) | 13 (46) | ns |
| *MAP rest (mmHg)* | 102.9 ± 9.9 | 97.5 ± 10.2 | 102.3 ± 13.2 | 102.1 ± 16.3 | ns |
| *MAP peak (mmHg)* | 145.4 ± 15.7 | 150.0 ± 17.0 | 148.1 ± 20.0 | 149.9 ± 18.8 | ns |
| *RPP peak* | 25,767 ± 7,494 | 27,601 ± 5,269 | 28,345 ± 6,641 | 28,181 ± 6,264 | ns |
| *RER peak* | 1.08 ± 0.07 | 1.08 ± 0.05 | 1.08 ± 0.07 | 1.09 ± 0.06 | ns |
| *VO_2_/work slope* | 10.8 ± 1.3 | 10.0 ± 1.2 | 10.3 ± 1.4 | 10.9 ± 1.2 | ns |
| *VO_2_ rest (mL/min/kg)* | 4.0 ± 1.3 | 4.5 ± 1.4 | 4.1 ± 1.3 | 4.7 ± 1.4 | ns |
| *VO_2_ peak (mL/min/kg)* | 18.9 ± 3.8 | 19.7 ± 3.7 | 18.8 ± 5.6 | 19.2 ± 4.3 | ns |
| *VO_2_ peak (%VO_2max_)* | 75.5 ± 16.0 | 80.5 ± 16.4 | 77.4 ± 13.1 | 81.3 ± 10.8 | ns |
| *VE/VCO2 slope* | 27.7 ± 3.6 | 28.0 ± 3.8 | 27.5 ± 4.7 | 28.8 ± 4.51 | ns |
| *O_2_ pulse peak (mL/bpm)* | 12.2 ± 2.8 | 12.4 ± 2.8 | 11.7 ± 2.8 | 12.2 ± 2.7 | ns |
| *O_2_ pulse peak (%VO_2_peak)* | 93.8 ± 19.8 | 96.6 ± 17.4 | 92.5 ± 14.6 | 95.6 ± 14.2 | ns |
| *AV O_2_ diff peak (mL/dL)* | 11.5 ± 3.0 | 11.6 ± 2.5 | 12.3 ± 3.7 | 12.5 ± 4.0 | ns |
| Echocardiography | | | | | |
| *EDVi (mL/m^2^)* | 52.0 ± 12.2 | 54.3 ± 12.4 | 51.0 ± 11.5 | 53.9 ± 10.2 | ns |
| *LVMi (g/m^2^)* | 89.9 ± 16.1 | 93.9 ± 22.1 | 89.2 ± 18.9 | 89.5 ± 17.1 | ns |
| *LAVi (mL/m^2^)* | 24.8 ± 8.4 | 25.3 ± 7.6 | 25.0 ± 6.8 | 25.6 ± 7.0 | ns |
| *CO rest, L/min* | 5.7 ± 1.6 | 5.7 ± 1.2 | 5.2 ± 1.2 | 5.7 ± 2.0 | ns |
| *CO peak, L/min* | 14.3 ± 4.2 | 14.2 ± 3.8 | 13.6 ± 4.3 | 14.0 ± 4.4 | ns |
| *LVEF rest (%)* | 60.5 ± 3.6 | 60.6 ± 4.1 | 58.1 ± 5.1 | 60.7 ± 5.7 | ns |
| *LVEF peak (%)* | 69.1 ± 4.7 | 68.6 ± 5.2 | 66.3 ± 6.9 | 68.4 ± 5.8 | ns |
| *ΔLVEF* | 8.6 ± 3.1 | 7.9 ± 4.1 | 8.2 ± 4.0 | 9.4 ± 2.6 | ns |
| *GLS rest (%)* | 17.3 ± 2.7 | 18.7 ± 2.7 | 15.8 ± 2.3 | 16.7 ± 2.8 | ns |
| *GLS 4 min (%)* | 19.2 ± 3.2 | 20.1 ± 2.9 | 17.7 ± 2.9 | 18.7 ± 3.1 | ns |
| *ΔGLS (%)* | 1.9 ± 1.8 | 1.4 ± 1.4 | 1.9 ± 1.3 | 2.0 ± 1.9 | ns |
| *S’ mean rest (cm/sec)* | 8.8. ± 1.7 | 9.0 ± 1.9 | 9.8 ± 2.0 | 9.7 ± 1.7 | ns |
| *S’ mean peak (cm/sec)* | 13.9 ± 2.9 | 14.1 ± 2.9 | 14.5 ± 2.5 | 14.4 ± 2.2 | ns |
| *ΔS’ mean* | 5.2 ± 2.3 | 5.2 ± 2.2 | 4.7 ± 1.7 | 4.7 ± 1.4 | ns |
| *E/e’ rest (cm/sec)* | 8.3 ± 2.2 | 7.7 ± 2.0 | 8.7 ± 2.7 | 7.8 ± 2.3 | ns |
| *E/e’ peak (cm/sec)* | 8.7 ± 1.8 | 8.2 ± 2.3 | 9.0 ± 2.4 | 8.6 ± 1.5 | ns |
| *SVR rest (dyne*sec/cm)* | 1,551 ± 442 | 1,404 ± 258 | 1,640 ± 362 | 1,475 ± 404 | ns |
| *SVR peak (dyne*sec/cm)* | 873 ± 206 | 880 ± 185 | 938 ± 273 | 923 ± 283 | ns |

**Additional file 2: Table S1.** Cardiopulmonary exercise test, 2D Echocardiograpy, Doppler, Tissue Doppler, and Speckle tracking parameters. P-value is the result of a two-point ANOVA for repeated measures.
